# A primary school active break programme (ACTI-BREAK): study protocol for a pilot cluster randomised controlled trial

**DOI:** 10.1186/s13063-017-2163-5

**Published:** 2017-09-19

**Authors:** Amanda Watson, Anna Timperio, Helen Brown, Kylie D. Hesketh

**Affiliations:** Institute for Physical Activity and Nutrition (IPAN), School of Exercise and Nutrition Science, Deakin University, Geelong, VIC Australia

**Keywords:** Physical activity, School, Classroom, Academic outcomes, Children, Intervention, Protocol

## Abstract

**Background:**

Levels of overall physical activity have been shown to decline across childhood. Schools are considered ideal settings to promote physical activity as children spend a large amount of their waking hours at school. Time-efficient physical activity strategies that demonstrate a positive impact on academic-related outcomes are needed to enable physical activity to be prioritised in the school day. The ACTI-BREAK programme requires classroom teachers to integrate active breaks; 5-min bursts of moderate-intensity physical activity into their classroom routine. Active breaks have been shown to be effective in improving academic-related outcomes, a potentially appealing aspect for teachers and schools. The primary aim of this study is to assess the feasibility and potential efficacy of the ACTI-BREAK programme on children’s academic achievement. Secondary aims are to explore the impact of ACTI-BREAK on children’s on-task behaviour and objectively measured physical activity levels.

**Methods:**

ACTI-BREAK is a 6-week, classroom-based, physical activity intervention. This pilot trial of the programme will be evaluated using a cluster randomised controlled design. Government primary schools in metropolitan Melbourne, Australia will be invited to participate in the programme in 2017. Randomisation will occur at the school level, with the aim to recruit six schools (three intervention and three control). The ACTI-BREAK programme is theoretically grounded, and was developed with input and guidance from current primary school teachers. Teachers from the intervention schools will receive a 45-min training session and be asked to incorporate ACTI-BREAKS into their classroom routine three times per day for 6 weeks. Intervention support will be provided via assisted delivery. The primary outcomes will be children’s academic achievement in mathematics and reading. Children’s on-task behaviour and school-day physical activity will be assessed as secondary outcomes. Process evaluation will also be carried out.

**Discussion:**

The ACTI-BREAK programme has been designed to be a time-efficient, feasible and appealing approach to physical activity promotion for schools. This study will assess required teacher time commitment and the potential for the ACTI-BREAK programme to improve academic-related outcomes and school-day physical activity levels with the potential for a full-scale trial in the future.

**Trial registration:**

Australia New Zealand Clinical Trials Registry, identifier ACTRN12617000602325. Retrospectively registered on 27 April 2017.

**Electronic supplementary material:**

The online version of this article (doi:10.1186/s13063-017-2163-5) contains supplementary material, which is available to authorized users.

## Background

While the health benefits of physical activity are well established [1], higher levels of physical activity are also associated with enhanced academic-related outcomes including cognitive function, classroom behaviour and academic achievement [2–4]. However, population based-studies have reported that over 50% of children in Australia and internationally are not meeting the recommended levels of physical activity, and rates of compliance decline with increasing age from the early primary school years [5–8]. Thus, increasing children’s levels of physical activity has important implications for both health and academic-related outcomes during childhood. The brain may be particularly sensitive to the effects of physical activity during pre-adolescence as the neural circuitry of the brain is still developing [9]. Thus, in order to stem age-related declines in physical activity and maximise academic-related outcomes, pre-adolescent children aged 8 to 10 years were selected as the target population for this study.

Primary schools can provide an ideal setting for the promotion of children’s physical activity due to the amount of time children spend in this setting [10]. However, allocating more time for physical activity during the school day can be problematic due to competing curriculum demands. Thus, to enable physical activity to be prioritised in the school day, time-efficient physical activity strategies that benefit academic achievement are needed [11]. One solution could be to break up sedentary time with light-intensity physical activity (e.g. through the use of standing desks). A recent (2016) systematic review investigated the impact of standing desk interventions within the classroom (*n* = 11 studies). While results showed that standing desks within the classroom were not detrimental to children’s academic-related outcomes (*n* = 3 studies) [12], more consistent positive associations have been observed for the effect of physical activity of at least moderate-intensity on academic outcomes [13]. Thus, combining breaks in sedentary time with moderate-intensity physical activity is hypothesised to have enhanced benefits on academic-related outcomes. Active breaks, requiring classroom teachers to integrate short bursts of physical activity into their classroom routine [14, 15], may provide such a more effective approach.

Active breaks have been shown to be effective in improving children’s academic achievement [16, 17], classroom behaviour (e.g. on-task behaviour) [14, 15, 18, 19], and cognitive function (e.g. attention) [20, 21]. Thus, active breaks can provide a potentially appealing physical activity promotion strategy for teachers and schools. However, there are several factors associated with their practical application. During qualitative interviews with teachers about their perceptions of using active breaks in the classroom, teachers explained the need for active breaks to cater for time (e.g. due to academic accountability) and space constraints [11, 22]. Thus, teachers preferred active breaks that were quick and easy to implement (i.e. require no set-up or equipment) and that were able to be performed within the limited available space in the classroom [11, 22]. Teachers also expressed concern that active breaks would have an adverse effect on classroom behaviour [11, 22]. Thus, time and space constraints should be considered when developing classroom-based active break programmes, and the potential for active breaks to improve on-task classroom behaviour should be highlighted during teacher training sessions.

There are several limitations with existing active break programmes. No evidence-based active break intervention to date has involved classroom teachers in the development of the intervention [23]. This has important implications for the feasibility and sustainability of such programmes outside of the research context [4]. Many existing programmes require active break durations of between 10 and 15 min [19, 21, 24–26]. However, qualitative interviews with teachers about their satisfaction with a previous active break programme indicated that active breaks longer than 5 min were unlikely to be adopted due to time constraints [27]. This discrepancy between evidence and practice highlights the importance of involving teachers in the development phase to ensure that interventions have real-world applicability.

While the existing evidence base suggests that active breaks need to last at least 10 min and be of at least moderate physical activity intensity in order to be effective for improving academic-related outcomes [15, 16], few studies (two interventions) have investigated the effect of active breaks shorter than 10 min on academic-related outcomes, and results were inconsistent [14–16, 20]. Further, the shorter duration active breaks in these interventions were implemented once per day. A recent (2016) study showed that children aged 10 to 13 years who performed two 20-min moderate-intensity active breaks per day had significantly better selective attention scores compared to children who performed one active break per day [28], highlighting the potential effectiveness of frequent active breaks. However, the impact of frequent, short (i.e. 5-min), active breaks on academic-related outcomes is unknown, and further research is needed to determine if this more feasible approach can be effective in improving academic-related outcomes.

A further limitation of previous research in this area is the choice of outcome assessment measures. Most studies have used standardised tests or grades to assess intervention effects on academic achievement [29–31]. Although helpful in assessing long-term impacts (i.e. yearly) these measures are not sensitive to short-term academic progress [32]. Given that most active break interventions have been implemented over relatively short durations (6 weeks to 8 months) [17, 30, 33], important intervention effects on academic achievement may have been missed. One way of assessing academic achievement over the short term is through curriculum-based measures [34]. Curriculum-based measures are commonly used by teachers to assess progress in key curriculum areas (e.g. mathematics and reading) over the short term [35]. These tools are sensitive to small changes in academic achievement, and can be administered frequently (e.g. weekly) [34] and therefore, may be a more appropriate measure for intervention periods of less than 1 year. It may be important for active break programmes to demonstrate positive effects on academic achievement, especially in the areas of mathematics and language (e.g. reading), as these test results are often used for the evaluation of schools [36].

Lastly, the majority of active break studies either did not measure intervention effects on physical activity, or used pedometer measures [31], which do not provide an accurate measure of physical activity intensity. To the authors’ knowledge, only one active break study has used an objective measure of physical activity intensity to determine effects on moderate- to vigorous-intensity physical activity levels [18]. Results showed that students in classrooms where teachers reported implementing active breaks in the past week were more likely to obtain 30 min per day of moderate- to vigorous-intensity physical activity during the school day (odds ratio (OR) = 1.75; *p* = 0.002) than children in classrooms where active breaks were not implemented [18]. However, that study measured physical activity on a subsample of participants only [18]; thus, results may not be generalisable to all children in the class. The ACTI-BREAK programme was developed to address limitations in previous studies including lack of teacher involvement in the development phase, the use of academic outcome assessment measures designed to assess long-term change in short-term interventions, and the lack of objective physical activity measurement.

### Aims

The primary aim of this study is to assess the feasibility and potential efficacy of a 6-week, pilot, classroom-based, physical activity intervention (ACTI-BREAK programme) on achievement in mathematics and reading in children in years 3 and 4 (aged approximately 8 to 10 years) attending primary (elementary) school in Melbourne, Australia. The impact of ACTI-BREAK on children’s on-task classroom behaviour and objectively measured school-day physical activity levels will be explored as secondary aims.

## Methods

### Study design

**Fig. 1:**
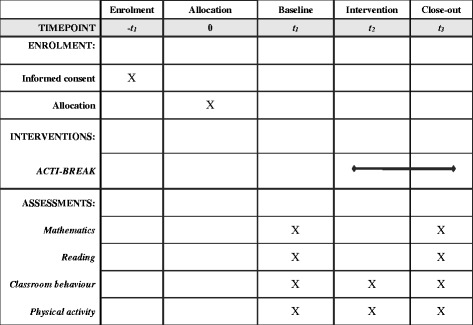
Standard Protocol Items: Recommendations for Interventional Trials (SPIRIT) Figure

**Fig. 2:**
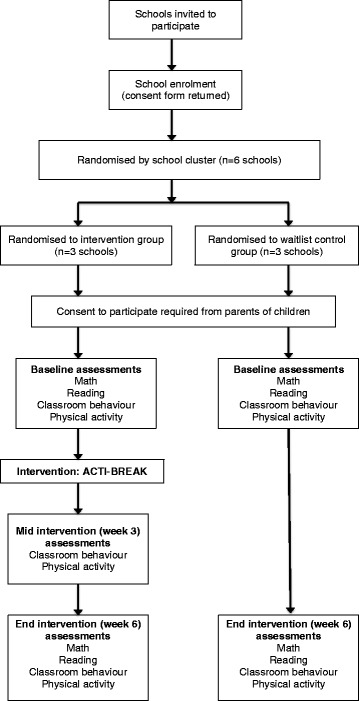
Flow diagram of participants through the ACTI-BREAK study

A pilot cluster randomised controlled trial will evaluate the ACTI-BREAK programme compared with a waitlist control. The design, conduct and reporting of the ACTI-BREAK programme will adhere to the Consolidation Standards of Reporting Trials (CONSORT) guidelines, and is guided by the Standard Protocol Items: Recommendations for Interventional Trials (SPIRIT) Statement. Additional file 1 shows the completed SPIRIT Checklist (see Additional file 1). Figure 1 provides an overview of the schedule for enrolment, interventions and assessments (SPIRIT Figure) and Fig. 2 shows a diagram of participant flow through the study. Principals, teachers, and parents will need to provide written informed consent to participate in the study. Ethical approval has been attained from Deakin University Human Research Ethics Committee, Melbourne, Australia (2016-020) and the Victorian Department of Education and Training (2016-002962).

### Power calculation

The detectable difference, based on grade-based benchmarks for year-3 reading scores in the Wheldall Assessment of Reading Passages (WARP) test [37] at mid-year, and 12 clusters (classes) per group, each with nine students, is 23.55 points in reading achievement with power set to 80%. Based on results from this study effect sizes will be generated to inform the sample size needed for a full-scale trial.

### Study setting

Government primary schools located within a 30-km radius of Deakin University and with a Victorian Socio-economic Indexes for Areas (SEIFA) Index of relative socio-economic advantage and disadvantage deciles of 4, 5, or 6, representing middle socio-economic position (SEP) will be invited to participate. Middle SEP government schools were chosen for this study as children attending these schools represent a large number of primary school children in Australia, making results likely to be generalisable to a large number of schools and children. Students attending Victorian government schools spend approximately 6.5 h per day at school (9.00 a.m. to 3.30 p.m.), and have a short recess/snack break (approximately 30 min) and a longer lunch break (approximately 1 h). The intervention will target year-3 and -4 classes (aged approximately 8–10 years). For schools to be eligible, they must have straight or composite year-3 and year-4 classes. Schools where composite classes mixed year-3 and year-4 students with other grades (e.g. years 2/3 or years 4/5 composites) were not eligible. Based on a 37% response rate (as demonstrated in a similar study conducted in the Australian school context [38]) and an average class size in Victorian schools of 23 students per class [39], we anticipate that nine students per class will consent to take part in this pilot study. For feasibility reasons, the intention is to recruit six schools (three intervention and three control) from which a sample size of 216 children is estimated (six schools × four classes (two classes per year level) [40] × nine students).

### Recruitment of schools

Principals from eligible schools will be invited to participate initially via email, and then contacted via telephone 1 week later. A researcher (AW) will meet with all interested principals to explain the requirements of study participation. Principals who agree to their school participating in the study will be provided with a plain language statement and consent form to be signed and returned prior to participation.

### Recruitment of participants

Once consent is obtained from school principals, written consent for participation will be obtained from teachers of year-3 and year-4 classes. Then, all year-3 and year-4 children at participating schools will be provided with an information pack containing a plain language statement and consent form to be given to their parents or guardians to provide consent for the child’s participation. As the school will have consented to the programme being delivered to all year-3 and year-4 children, and the programme will be delivered by classroom teachers as part of their daily classroom activities, consent from parents will only be required for the evaluation components of the study. Thus, all children in participating classes will join in the ACTI-BREAKS; however, data will only be collected from children with parental consent as part of the evaluation of ACTI-BREAKS. Further, so that no child feels excluded, all children in participating classes will be invited to participate. However, data from children with diagnosed behavioural or learning problems (e.g. attention-deficit hyperactivity disorder (ADHD)) will be excluded from analyses. The schools, teachers and children will not be paid to participate in the ACTI-BREAK programme.

### Randomisation

Schools will be randomised at the school level (to avoid potential for contamination) to either the intervention or waitlist control group, prior to baseline assessments. The waitlist control group will be provided with the intervention materials after the final data collection period. Randomisation will be carried out via computer-generated random number sequence by a researcher who has no contact with the schools or participants. As all schools will be from middle socioeconomic position areas within a similar geographical location, it is unlikely for there to be large differences in baseline characteristics, including academic achievement levels. Any differences between the intervention and control groups will be by chance [41], and adjusted for in the analyses.

## Intervention

### Development

The ACTI-BREAK programme was informed by a review of the relevant academic literature and consultation with current primary school teachers. This study embeds the Capability, Opportunity, Motivation-Behaviour (COM-B) model [42], and is underpinned by Social Cognitive Theory (SCT) [43] and the Ecological Model (EM) (see Table 1). Nine teachers from schools demographically similar to the intervention schools were interviewed about the feasibility of introducing regular active breaks into primary school classrooms. Of those teachers, most (*n* = 6) had experience using active breaks in their classroom. Topics covered included preferred duration, intensity and frequency of active breaks, as well as potential barriers and facilitators to implementation. Teachers considered frequent (multiple times per day), 5-min active breaks feasible, and anything longer than 5 min was deemed unlikely to be adopted by teachers. Further, teachers preferred moderate-intensity as opposed to vigorous-intensity active breaks which they considered to be disruptive (due to students needing to get drinks and take off sweaters). Given this and the support for the role of moderate-intensity active breaks for improving academic-related outcomes in the academic literature [19, 21, 28], the ACTI-BREAK activities were selected to be of moderate physical activity intensity. During the programme development phase, teachers also communicated that it would be important to have a range of different active break activities from which they could select activities that best engaged their class and to allow for variety. Thus, the actual activities undertaken will differ by class; however, all activities are designed to be of moderate intensity. An important element of the ACTI-BREAK programme is that the activities do not require any set-up and only use equipment already available in classrooms (e.g. music), as in the programme development consultation teachers consistently expressed the need for active breaks to be quick and easy to adopt. Lastly, teachers stated that the scheduling of ACTI-BREAKS will need be determined by individual teachers due to variations in timetables across schools and classes (e.g. due to specialist subjects).

**Table 1:** Theoretical basis of the ACTI-BREAK programme

Constructs	Behaviour-change technique	Example in intervention
During teacher training		
Individual		
*Skills/capability*	* Demonstration of behaviour * Practice behaviour * Provide feedback	* During the teacher training session and assisted rollout phase the intervention teachers will be provided with demonstrations of a range of ACTI-BREAK activities * Researcher assisted rollout during weeks 1 and 2 of the intervention
*Outcome expectations*	* Provide information	* Teacher training session provides information on academic-related benefits of active breaks
*Support*	* Information about others’ approval	* Share anonymous feedback from teachers who have used active breaks in their classroom obtained during the qualitative intervention development phase (teacher interviews)
During intervention		
Individual		
*Enjoyment (students)*	* Provide opportunities to participate in enjoyable physical activities	* The ACTI-BREAK programme provides a variety of different active break options for teachers and/or students to select from
*Goals*	* Goal setting	* Set goal to deliver 3 × 5-min ACTI-BREAKS per day
*Monitoring*	* Self-monitoring of behaviour	* During visits to schools (to fit and collect activity monitors and carry out academic assessments), teachers will be prompted to complete the teacher log after each ACTI-BREAK
Interpersonal		
*Social support*	* Provide general encouragement	* During visits to schools to fit and collect activity monitors and carry out academic assessments
Environmental		
*Time*	* Minimising time barrier	* All ACTI-BREAKS are quick and easy to implement
*Prompts*	* Prompts/cues	* A log will be placed on the whiteboard to remind teachers to do ACTI-BREAKS, and teachers will ask students to hold them to account * ACTI-BREAK activities will be printed on individual cards

### The ACTI-BREAK programme

The ACTI-BREAK programme will involve teachers implementing 5-min active breaks, three times daily in their classrooms for 6 weeks, using activities adapted from a variety of sources [44–47]. Permission to use and modify these activities was sought and attained from the authors (personal communication). The ACTI-BREAK activities are age-appropriate and include a variety (*n* = 30) of active break options with suggested modifications for easier or more challenging activities. ACTI-BREAK activities incorporate drama (e.g. jog on the spot as if a big scary bear is chasing you), games (e.g. musical chairs), following instructions (e.g. when the music stops, touch 10 chairs in a row), and technology/websites (e.g. GoNoodle and YouTube). Examples of ACTI-BREAK activities can be found in Table 2. A 6-week trial was chosen for the initial assessment of programme feasibility based on pragmatic reasons as it allows the trial to be completed within a single school term (including teacher training and baseline assessments pre trial, as well as teacher and student qualitative feedback post trial). Further, the academic literature shows that intervention periods as short as 3 weeks may be sufficient duration to see benefit to academic-related outcomes [14, 20].

**Table 2:** Example activities from the ACTI-BREAK programme

ACTI-BREAK	Description
I wonder if…?	The teacher says ‘I wonder if…?’ And the students respond, ‘What do you wonder?’ The teacher then specifies a movement and the students perform that movement until the teacher says ‘I wonder if…?’ again. For example, ‘I wonder if students can walk backward without touching anyone or anything?’ [44]
As if	The teacher reads a sentence to the class, and students act out each sentence for 20 to 30 s, e.g. ‘jump in place *as if* you’re popcorn popping’ [47]
GoNoodle ‘Guided Dancing’	Go to the GoNoodle website and select the ‘Guided Dancing’ link, and select a video. Students follow along to the characters on the screen. Some options include ‘The Maxarena’ (*Macarena*), ‘Happy’ (from *Despicable Me*) and ‘The Continental Drift’ (Sid shuffle) from *Ice Age* [45]

To improve adherence to the intervention protocols, the intervention will be implemented using assisted delivery. Week 1: a researcher (AW) and the classroom teacher will implement the ACTI-BREAKS together; week 2: the classroom teacher will implement the ACTI-BREAKS with the researcher observing and providing feedback; week 3 onwards: the classroom teacher will deliver the ACTI-BREAKS on their own with the researcher not present. The researcher will provide teachers with general encouragement and support during visits to schools to fit and collect activity monitors and carry out academic assessments. Further, teachers will be able to email or call the researchers at any time if they have concerns or require further support.

#### Teacher training session

At each intervention school, all teachers of years 3 and 4 will participate in a one-off 45-min face-to-face training session approximately 1 week prior to implementing the ACTI-BREAK programme. The session will be conducted on school grounds at a convenient time for teachers, and will be delivered by the researcher (AW) who is a qualified and experienced primary school teacher. The training session is designed to inspire and equip the teachers with the necessary skills and knowledge to be able to implement the ACTI-BREAK programme in their classrooms. This training session will include a rationale for adding active breaks to the classroom routine, current research evidence highlighting the potential positive effect of active breaks on academic-related outcomes, explanation of the ACTI-BREAK programme and what they are required to do, and demonstrations of a range of the ACTI-BREAK activities. However, as the researcher will be delivering and observing the ACTI-BREAK programme during the first 2 weeks of implementation, extensive demonstrations of the active break activities will not be provided during the training session. At the completion of the training session, teachers will be provided with intervention materials including the ACTI-BREAK programme prompt cards, and classroom timers.

### Primary outcomes

The primary outcome of academic achievement will be assessed using measures of reading and mathematical achievement. These assessments were chosen as the primary outcomes for this study as these are the two key pillars by which teachers and schools assess academic progress [36].

#### Reading achievement

Reading achievement will be assessed using the WARP test [37]. This curriculum-based measure, designed for the Australian school context, is designed to track reading fluency of students in years 2 to 5, and involves children reading for 1 min from a 200-word passage [37]. The number of words read correctly in that minute is a measure of the student’s level of reading fluency [37]. Reading fluency has been shown to be a powerful predictor of reading performance, with meta-analytic results showing a strong correlation between curriculum-based measures of reading fluency and other standardised tests of reading performance (*r* = 0.67) [48]. The WARP test includes a set of three initial assessment passages and a set of 10 progress-monitoring passages [37]. On advice from the instrument author (personal communication), the progress-monitoring passages were chosen for the current study, as they can be expected to be sensitive to change over the 6-week intervention period. The 10 progress-monitoring passages correlate very highly to each other in terms of reading difficulty (*r* = 0.95–0.98) [49]. Thus, to eliminate potential learning effects, a different passage will be administered at baseline and end of the intervention, and is chosen based on advice from the author (personal communication). A researcher with a primary school teaching qualification and experience will administer this test individually to each student with parental consent, at baseline and during the final week (week 6) of the intervention.

#### Mathematics achievement

Achievement in mathematics will be assessed using the One-Minute Tests of Basic Number Facts, reproduced with permission from the Australian Council for Educational Research [50]. This tool consists of four One-Minute Basic Number Facts Tests [50], and is designed for the Australian school context. Each test has 33 items that focus on one of each of the following number operations: addition, subtraction, multiplication, and division [50]. This test has good test-retest reliability (0.88 to 0.94 depending on age level) [50]. To ensure alignment with the year-3 and year-4 curriculum early in the school year, and, therefore, potential to observe improvement, the subtraction test was chosen for this study. The addition test was not chosen due to the potential for ceiling effects, and thus limited potential to observe improvement. A researcher with a primary school teaching qualification and experience will administer this test to the whole class at baseline and at the end (week 6) of the intervention. Only data from students with parental consent will be used in the analyses. To accommodate class availability (e.g. due to specialist subjects) the reading and mathematics assessments will be carried out at a time that suits each classroom teacher.

### Secondary outcomes

#### On-task classroom behaviour

Information will be collected on student on-task classroom behaviour at the individual level for those students with parental consent, and at the whole class level (no identifying information will be retained). As classroom behaviour can vary as a function of time of day, each class teacher will be required to record all observations of classroom behaviour at a consistent time each day. However, due to differing timetables across classes and schools, it will not be possible for all teachers to record behaviour observations at the same time of day.

Teachers at intervention and control schools will be required to observe behaviour during a 10-min observation period during a seated lesson at three time points at baseline and again at the end (week 6) of the intervention. In addition to baseline and the end of the intervention observations of behaviour, teachers at intervention schools will be required to record observations of behaviour during a 10-min observation period immediately before and immediately after participation in an ACTI-BREAK at three time points during week 3 of the intervention. This pre-post measure will enable the acute effect of active breaks on classroom behaviour to be explored. Teachers will record observations of individual and group classroom behaviour simultaneously in order to limit the number of times that teachers will be required to record observations. Teachers will be given a record sheet with student names pre-filled, and each observation will take only a few seconds to complete. Given this, and as teachers continually monitor classroom behaviour as part of their daily routine, this approach is considered feasible. Lastly, teachers’ reports of classroom behaviour as reported in a similar previous study were shown to be reliable (alpha = 0.85) [18]; however, to the authors’ knowledge only one previous study has used such measures. Nonetheless, although the same class teachers who deliver the intervention will also record observations of behaviour, risk of bias is expected to be minimal.

Classroom behaviour at the individual level will be measured by teachers using a tool adopted from the Direct Behaviour Rating Scale [51]. The Direct Behaviour Rating Scale is a hybrid of direct observation and behaviour rating scales. This observation tool requires teachers to indicate for each child, on a scale from 1 (0%) to 10 (100%), the percentage of time that they are on-task, referred to as academically engaged in this tool (i.e. listening to the teacher, writing, looking at instructional materials, etc.) during the observation period. This tool provides a valid measure of classroom behaviour, when compared with direct observation (*r* = 0.81 to 0.87) [52]. Further, this tool has evidence of reliability (*r* = 0.91) based on data from 617 primary school students obtained from 44 classroom teachers [51].

Behaviour at the whole class level will be measured using a modified version of the Classroom Behaviour and Assets Survey-Teacher which is designed to efficiently provide a snapshot of the teacher’s perception of the behaviour of a whole class of students [53]. Teachers will be required to indicate the proportion of the class displaying on-task behaviour (as defined in the individual behaviour assessment tool), during the observation period. Response options include: 0 (0 students), 1 (1–2 students), 2 (a few students), 3 (about a quarter of the class), 4 (about half of the class), 5 (about three quarters of the class), 6 (most of the class), and 7 (all of the class) [53]. Although few tools are available for assessing classroom behaviour at the classroom level, and information on reliability and validity is lacking, a modified version of this tool has been used in a similar study, with evidence of reliability (alpha = 0.85) [18].

#### Physical activity

Waist-worn ActiGraph GT3-X accelerometers (children with parental consent) will be used to provide an objective measure of children’s moderate- to vigorous-intensity physical activity across the school day. The ActiGraph accelerometer is commonly used in studies involving children [54] and has documented evidence of validity and reliability for measuring children’s physical activity [55]. Accelerometers will be worn during waking hours for seven consecutive days at baseline, mid-intervention (intervention group only), and the end (week 6) of the intervention period. Measures will be taken in the last week of the intervention, rather than post intervention as the intervention is intended to have acute effects.

The accelerometers will be distributed and collected from the child’s school at the beginning and end of each 7-day wear period. The researcher will explain about, and fit the monitors to children with parental consent and provide children with an information leaflet to take home to their parents explaining when and how the monitor should be worn and care instructions. Participants’ physical activity will be included for analysis if they wear the accelerometer for at least five school hours on at least one school day, as used in similar studies, involving physically active lessons [23]. Freedson cut-points will be used to classify physical activity as moderate- to vigorous-intensity [56]. These cut-points have been shown to accurately classify children’s moderate- to vigorous-intensity physical activity (ROC-AUC = 0.90) [57]. Data will be collected in 15-s epochs [58] and non-wear time will be defined as 20 min of consecutive zero’s, as commonly used in studies involving children [59].

### Intervention fidelity

To assess fidelity of implementation, children will wear accelerometers mid-way through the intervention (week 3) as well as during the final week of the intervention period (week 6). Furthermore, teachers will complete a log of the ACTI-BREAKS that they complete over the intervention period. The researcher (AW) will collect these at the end of every week to ensure teacher fidelity throughout the study. Accelerometer data will be compared with teacher logs of ACTI-BREAKS completed at week 3 and week 6 of the intervention to verify that students were physically active at a moderate intensity for the 5-min ACTI-BREAK duration.

### Process evaluation

Subjective evaluations of the intervention components will also be provided by students and teachers throughout the intervention. During each of weeks 1, 3, and 6 of the intervention, students will be asked to rate three different ACTI-BREAK activities (decided by the classroom teacher), immediately following participation using a 4-point Likert scale ranging from ‘I hated it’ = 1 to ‘I loved it’ = 4. Upon competition of the 6-week programme, students will be asked to complete a questionnaire to rate how they feel about the ACTI-BREAK activities using a 4-point Likert scale with responses ranging from ‘strongly disagree’ = 1 to ‘strongly agree’ = 4 in the areas of (1) enjoyment, (2) effect on learning and behaviour, (3) ability to do the activities, and (4) preferred duration, intensity, and frequency of activities. For example, ‘I enjoyed the ACTI-BREAK programme’ and ‘I found it easier to concentrate after doing ACTI-BREAK activities’. Students will also have the opportunity to provide additional feedback on the following open ended questions: ‘What did you like about the ACTI-BREAK programme?’ and ‘Was there anything you didn’t like about the ACTI-BREAK programme?’

Post-intervention focus groups will also be conducted with students, facilitated by the researcher. The focus groups will be recorded and later transcribed, with participant consent. Specifically, the questions asked will be designed to provide a more in-depth insight into topics covered in the questionnaire. For example, students will write down the names of two of their favourite, and two of their least favourite ACTI-BREAK activities, and then explain what they liked or did not like about the particular ACTI-BREAK, and what they would change.

A one-on-one 30- to 45-min telephone interview will be conducted with the teachers involved in the intervention group, after programme completion. The researcher will conduct these interviews which will be recorded and transcribed. The interviews with teachers will be designed to identify barriers and facilitators to implementation of ACTI-BREAKS, as well as strengths and weaknesses of the programme. The following types of questions will be asked, for example: ‘Were there any ACTI-BREAKS you thought worked particularly well/didn’t work particularly well?’ and ‘Was there anything that made it difficult for you/helped you to use the ACTI-BREAK programme?’

### Data analysis

All statistical analysis will be conducted using Stata 14.0. Multilevel mixed-effects linear regression models will be used to assess the impact of group (intervention vs. control) on achievement scores in reading and mathematics, mean per cent of time in on-task behaviour, and physical activity level at the end of the intervention, each adjusted for baseline levels of the corresponding variable and clustering by class. All analyses will control for physical activity levels at baseline. The fixed effect of school will be added to the model to account for the unit of randomisation. All analyses will be stratified by sex. Alpha levels will be set at *p* < 0.05. As this is a pilot study, the intervention will not be adequately powered to detect small changes between groups. Instead, trends in associations will be investigated. Analyses will be conducted both on the intention-to-treat approach, and per protocol analysis (using data from fidelity checks).

Process evaluation data from focus groups and interviews will be analysed using Braun and Clarke’s six phases of thematic analysis [60]. Codes will be generated, and continually revised throughout the analysis process. Following coding of the transcripts, themes will be identified and defined. These data, along with data obtained from student enjoyment and satisfaction questionnaires, will provide information that can be used to improve the programme for future testing.

## Discussion

The primary aim of this study is to examine the potential efficacy of a classroom-based physical activity programme known as ACTI-BREAK on children’s achievement in mathematics and reading. The impact of ACTI-BREAK on children’s on-task behaviour and objectively measured school-day physical activity levels will be explored as secondary aims. Given that the programme is designed to be delivered by trained classroom teachers in the ‘real-world’ context, assessment of feasibility and fidelity will be a key feature of the pilot study.

A strength of this intervention is that it was developed with input and guidance from current primary school teachers to limit discrepancy between evidence and practice observed in previous studies [4]. For example, our interviews with teachers indicated that anything longer than 5 min would be unlikely to be adopted. Many existing active break programmes require active break durations of between 10 and 15 min [19, 21, 24–26]. However, longer duration active breaks may be unlikely to be feasible beyond the study setting [61].

An additional strength of this study is the use of a curriculum-based measure to assess achievement in reading and mathematics. These tools are sensitive to small changes in academic achievement, and can be administered frequently (e.g. weekly) [62, 63]. Many existing studies have had short intervention periods (less than 1 year) and used standardised tests to assess intervention effects on academic achievement [29, 30]. These tests are designed to be administered at least 1 year apart [32], and, therefore, important intervention effects on academic achievement are unlikely to be observed. The use of a curriculum-based measure in the current study provides a more suitable measure of academic achievement for the 6-week intervention period, making short-term progress in academic achievement more likely to be observed.

In addition to choice of academic outcome measure, the use of an objective measure of physical activity intensity and cluster randomised controlled trial study design are further strengths of this study. To the author’s knowledge, one previous active break intervention has used an objective measure of physical activity intensity [18]. However, that study relied on teacher reports of active break implementation in the past week (to compare physical activity levels of implementers vs. non-implementers) and intervention effects on physical activity levels were assessed on a subsample of participants only [18]. Nonetheless, results from that study highlight the potential for active breaks to contribute to increased physical activity across the school day [18].

Results from this study will provide insight into the feasibility of introducing frequent, short, moderate-intensity active breaks into classroom routines, given potential challenges in their application. Further, this study will explore whether this approach will provide enhanced benefits on academic-related outcomes, compared with breaks in sitting time.

### Limitations

Given that some of the teachers who participated in the intervention development referred to using active breaks in their classrooms previously, it is possible that some teachers in the control schools may also do active breaks in their classrooms. However, teachers in the intervention development mainly referred to ad-hoc stretches and coordinative exercises (e.g. Brain Gym); thus, any active break activities conducted by teachers in control schools are not likely to be as frequent or intense as those prescribed in the ACTI-BREAK programme. A further potential limitation relates to the use of teacher-reported observations of classroom behaviour. These observations will be recorded by the same teachers who will be delivering the intervention and thus have potential for bias. However, to the authors’ knowledge, only one previous study has used such measures which were shown to be reliable (alpha = 0.85) [18].

## Conclusions

This study has the potential to enhance key educational outcomes (e.g. reading and mathematics achievement) which may encourage teachers and school administrators to provide more opportunities for children to be active at school through incorporating short, active breaks into the classroom routine. The ACTI-BREAK programme has been designed to be a time-efficient, feasible and appealing approach to physical activity promotion in schools. This study will assess required teacher time commitment, the potential for the ACTI-BREAK programme to improve academic-related outcomes and physical activity levels, and its acceptability to teachers and students, with the potential for a full-scale efficacy trial in the future.

### Trial status

The trial is currently recruiting.

## Additional file

Additional file 1: Completed SPIRIT Checklist. (DOCX 64 kb)
